# P02.32. Complementing the homeostasis model by a Rhythm Model – a conceptual framework to understand non-pharmacological complementary therapies

**DOI:** 10.1186/1472-6882-12-S1-P88

**Published:** 2012-06-12

**Authors:** D Cysarz, C Scheffer, F Edelhäuser

**Affiliations:** 1 University of Witten/Herdecke, Herdecke, Germany

## Purpose

In recent years, many non-pharmacological complementary therapies have been investigated with respect to cardiovascular regulation, e.g. analysis of heart rate variability. Such therapies often show a specific impact on cardiovascular regulation. However, the homeostasis model does not enable a full understanding of these specific effects. Homeostasis postulates constancy of physiological functions to maintain basic functions of the organism. It is conceptually described by closed loops with negative feedback. If the single elements of the loops are known, then this model permits focused interventions (e.g. pharmacological inhibition or stimulation) in the context of a disease condition.

## Methods

A rhythm model is introduced that complements the homeostasis model. The model explicitly takes into account the spontaneous rhythms of the human organism. It is exemplified by recent findings in complementary speech therapy.

## Results

Non-pharmacological complementary therapies can be explained adequately with this model because these therapies often trigger systemic responses of the organism. With the help of rhythmical stimulation, they activate the regulation of several physiological functions. This leads to a re-tuning of the regulation if the therapy is repeatedly applied several times.

## Conclusion

The rhythm model is able to explain therapies that re-tune the regulation of physiologic functions. Furthermore, this model shows that rhythms and temporal structures of physiological functions are basic properties of living systems. A prediction of the effects imposed by non-pharmacological complementary therapies stimulating regulation is possible.

